# Micronuclei frequencies in lymphocytes and cervical cells of women with polycystic ovarian syndrome

**DOI:** 10.4274/tjod.10734

**Published:** 2017-09-30

**Authors:** Rengin Karataylı, Ayşe Gül Zamani, Kazım Gezginç, Ebru Tuncez, Sema Soysal, Fikriye Karanfil, Aynur Acar, M. Selman Yıldırım

**Affiliations:** 1 Konya Private Selçuklu Hospital, Clinic of Obstetrics and Gynecology, Konya, Turkey; 2 Necmettin Erbakan University Meram Faculty of Medicine, Department of Medical Genetics, Konya, Turkey; 3 Necmettin Erbakan University Meram Faculty of Medicine, Department of Obstetrics and Gynecology, Konya, Turkey; 4 Şanlıurfa Pediatric Disease Hospital, Clinic of Medical Genetics, Şanlıurfa, Turkey; 5 Necmettin Erbakan University Meram Faculty of Medicine, Department of Medical Education and Informatics, Konya, Turkey; 6 University of Health Sciences, Konya Training and Research Hospital, Clinic of Obstetrics and Gynecology, Konya, Turkey; 7 İstanbul Bilim University Faculty of Science, Department of Molecular Biology and Genetics, İstanbul, Turkey

**Keywords:** Micronucleus tests, polycystic ovarian syndrome, cervical smears, Lymphocytes, genotoxicity tests

## Abstract

**Objective:**

The aim of this study was to determine micronucleus (MN) frequencies in exfoliated cervical cells and peripheral blood lymphocytes of women with polycystic ovarian syndrome (PCOS).

**Materials and Methods:**

Fifteen patients with PCOS and 11 healthy control patients were included in the study. Cervical smears and peripheral blood were collected from all patients. Specimens were analyzed for MN frequencies and compared between the groups. In addition to MN, other nuclear anomalies connected with both genotoxicity and cytotoxicity were evaluated.

**Results:**

The MN frequencies in cervical smear and peripheral blood lymphocytes were compared in patients with PCOS and normal controls. There was no statistically significant difference between the groups regarding micronucleus frequency in peripheral blood lymphocytes (p=0.239). The mean MN scores in exfoliated cervical cells of patients with PCOS and normal controls were 1.19±0.57 and 0.74±0.34, respectively. The difference regarding micronucleus frequencies in cervical cells was statistically significant between the groups (p=0.032).

**Conclusion:**

Although study group is small, our study results support that there is an increased micronucleus frequency in cervical exfoliated cells of PCOS patients; this is a determinant of genetic hazard in the disease.

## PRECIS:

By using micronucleus (MN) genotoxicity tests, we determined MN frequencies in exfoliated cervical cells and peripheral blood lymphocytes of women with polycystic ovarian syndrome.

## INTRODUCTION

Polycystic ovarian syndrome (PCOS) is a common endocrinopathy in women and it is characterized by chronic oligoanovulation and clinical/biochemical hyperandrogenism. Its prevalance is reported to be around 3.5-10% regarding diagnostic criteria^(1)^.

Micronucleus (MN) is cytoplasmic chromatin condensations that appear as small nuclei, which are secondary to chromosomal fragmentation at the anaphase stage of cell division. MN is one of the established genotoxicity biomarkers in human erythrocytes, lymphocytes, reticulocytes, and exfoliated mucosa cells. Spontaneous or baseline MN frequencies in cultured human lymphocytes and exfoliated cells provide an index of accumulated genetic damage occurring during the life span of these cells. An increased frequency of MN is used as a measure to detect clastogenicity and aneugenicity. MN presence in cells reflects structural and/or numerical chromosomal aberrations arising during mitosis^(2)^. The genetic basis of PCOS has been investigated in several studies, and there is evidence of the presence of multiple gene polymorphisms, oxidative stress, and environmental factors in the background^(3,4)^. In patients with PCOS, increased oxidative stress and decreased antioxidant capacity have been reported, and those were all reported to be related to genetic damage in PCOS^(5)^.

Yesilada et al.^(6)^ reported that the MN frequency in peripheral blood lymphocytes of women with PCOS was increased. In our study, we aimed to investigate the micronuclei frequency both in peripheral lymphocytes and cervical squamous cells of patients with PCOS in order to demonstrate genetic damage, also in cervical smears.

## MATERIALS AND METHODS

The study was conducted Necmettin Erbakan University Meram Faculty of Medicine, Department of Gynecology, and Department of Medical Genetics between January 2012 and August 2012. Fourteen patients with PCOS and 11 controls with similar age and body mass index were recruited. PCOS was diagnosed using the Rotterdam criteria established in 2003. Patients with diseases related to hyperandrogenism such as hyperprolactinemia, Cushing’s disease, androgen-secreting tumors, and late-onset congenital adrenal hyperplasia were all excluded by their medical history and specific tests. All participants answered a modified version of the questionnaire of the Commission for Protection against Environmental Mutagens and Carcinogens^(7)^. Information about contraceptive methods used, histories of sexually-transmitted diseases, and the patients’ habits (smoking, drug use and numbers of sexual partners) were obtained. None of the patients included in the study had any systemic disease or history of smoking. Patients with multiple partners, abnormal cervical cytology results or history of genital warts were excluded from the study. All patients in the study had at least one negative smear test previously.

Informed consent was taken from all patients and the study was approved by Necmettin Erbakan University Meram Faculty of Medicine Research Ethics Committee (approval number: 2012/21).

### Sampling and scoring of exfoliated cervical cells

Exfoliated cervical cells were collected using brushes and transferred to Falcon tubes containing 0.9% serum physiologic for MN tests. The material was centrifuged and the supernatant was discarded, leaving the exfoliated cells in the pellet. Cells were treated with hypotonic solution for 5 min. They were then treated twice with 5 mL of freshly prepared cold methanol: acetic acid (3:1). Drops of the material were placed on cold damp slides and allowed to dry. Samples were stained using 5% Giemsa for 5 minutes.

**Figure 1:**
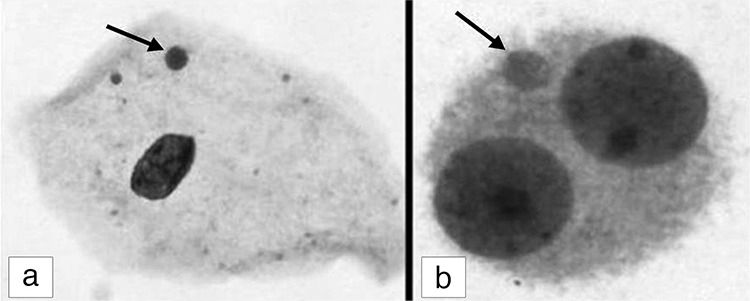
Micronucleus (arrow) in exfoliated cervical cell (a) and peripheral blood lymphocyte (b)

The slides were analyzed and 1000 epithelial cells were counted at a magnification of 1000 x (objective = 100 x with eyepiece = 10 x). Micronuclei were determined according to the criteria of Stich and Rosin^(8)^. Within the samples, only separate cells, without overlapping or folding, were analyzed. Micronuclei were counted if the structures had regular borders and were located inside the cytoplasm, with an intensity of staining less than or equal to that of the main nucleus and a size less than two-thirds of the size of the main nucleus (Figure 1a). The frequency of cells with micronucleus, binucleated cells (BNC), and cells with buds were reported as results.

### Sampling and scoring of peripheric blood lymphocytes

Heparinized blood samples were obtained. Lymphocyte cultures were performed for each subject and incubated for 72 h at 37 °C. According to the cytokinesis-block human lymphocyte MN test, cytochalasin-B (Sigma) was added after 44 hours into the final concentration of 3 Gg/mL^(9,10)^. After a total of 72 h culture, cells were harvested. First, they were treated with prewarmed hypotonic solution (0.075 M KCl) for a few minutes at room temperature and then resuspended twice in cold fresh fixative (methanol:glacialacetic acid, 3:1). Fixed cells were dropped on clean slides. After air drying the slides, they were stained using 5% Giemsa for 5 min. For each sample, 1000 BNC were observed to assess the frequencies of MN (Figure 1b). The cells scored for MN had to be clearly seen as binucleate. The number of MN in each binucleate cell was scored^(8)^. MN were accepted only when (i) they were separated from the main nuclei, but included within the corresponding cytoplasm, (ii) they had a chromatin structure similar to that of the main nuclei, (iii) they were coplanar to the main nuclei^(11)^, and (iv) they were no greater than one third the volume of the main nuclei^(10,12)^.

### Statistical Analysis

Statistical analysis was performed using SPSS for Windows, version 18.0 (SPSS Inc., USA). Continous variables are expressed as mean ± standard deviation. Student’s t-test was used to analyze statistical differences. The level of p<0.05 was considered statistically significant.

## RESULTS

The mean age in PCOS group was 29.3±5.2 years (range, 19-39 years), and in the control group it was 27.9±5.1 years (range, 21-35 years). The mean gravidity and parity in the PCOS group were 1.7±1.4 (range, 1-5) and 1.3±1.1 (range, 1-4) respectively, whereas they were 2.5±1.2 (range, 0-4) and 1.8±1.2 (range, 0-4) in the control group. There were no statistically significant differences between the groups regarding age, gravidity, and parity. The body mass indices were 25.1±3.4 kg/m^2^ (range, 21-31 kg/m^2^) in the PCOS group and 23.8±2.8 kg/m^2^ (range, 21-28 kg/m^2^) in the control group, respectively, without any statistical significance (p=0.147). The mean interval of coits was 4.7±3.6 in the PCOS group, whereas it was 5.1±3.1 in the control group; the difference was not statistically significant between the groups. The study results are summarized in Table 1. The MN frequency in the peripheral blood lymphocytes of women with PCOS was 10.93±6.5 (per 1000 cells), whereas it is 8.4±3.8 (per 1000 cells) in the control group. There was no statistically significant difference between the groups regarding MN frequency in peripheral blood lymphocytes (p=0.239). On the other hand, the MN frequency in cervical exfoliated cells was 1.19±0.57 (per 1000 cells) in PCOS group, whereas it is 0.74±0.34 (per 1000 cells) in the control group. The difference regarding MN frequency in cervical cells was statistically significant between the groups (p=0.032). In addition, other cellular discrepancies such as BNC and budding cells were 1.08±0.27 (per 1000 cells) and 0.10±0.08 (per 1000 cells) in the PCOS group, respectively, and 0.81±0.23 (per 1000 cells) and 0.06±0.06 (per 1000 cells) in the the control group. The difference between the groups regarding BNC was statistically significant (p=0.016), whereas the difference concerning budding cells was not statistically significant (p=0.147) (Table 2).

## DISCUSSION

PCOS, a common endocrinopathy among reproductive age women, is accepted to be associated with metabolic syndrome. It is also associated with severe long-term hazards such as type-2 diabetes, cardiovascular diseases, and endometrial cancer. Nowadays, PCOS and its relation with cancer is of special interest in scientific research. Oxidative stress due to an imbalance between the generation of reactive oxygen species and antioxidant defense has been investigated in the etiology of cancer development^(13,14)^. The imbalance between pro-oxidants and antioxidants has been proposed to be associated with reproductive diseases such as endometriosis, PCOS, and unexplained infertility. In the literature, there are various reports about increased oxidative stress and related comorbidity in patients with PCOS^(5,15,16)^. It is known that proteins, lipids, and DNA are exposed to damage as a result of oxidative stress. Moreover, in a study by Simic^(14)^, it was defined that oxidative stress was linked to chromosomal breakage and carcinogenesis. Zuo et al.^(17)^ reported that variable oxidative stress in PCOS, instability of genes, and DNA mutations increased the risk and potentially contributed to the pathogenesis of gynecologic cancers. Deepika et al.^(18)^ reported that serum malondialdehyde levels of oxidative stress markers showed a positive correlation with MN in patients with PCOS.

There are also studies about the increased oxidative stress markers in patients with PCOS and this mechanism has been accepted to be related to mutagenesis in these patients^(5,15)^. Some investigators reported a positive correlation between oxidative stress and lipid peroxidation and genotoxicity^(16,17,18)^. This type of damage can lead to chromosomal losses and rearrangements. MN are DNA-containing particles that occur during mitosis and result from unrepaired DNA double-strand breaks, leading to chromatin fragments or whole chromosomes being distributed incorrectly. Therefore, the MN test shows genetic damage that cells accumulate throughout life. At the molecular level, this kind of DNA damage could be silenced tumor supressor genes and activated oncogenes. It could then initiate cancer development by adding epigenetic changes^(17,18,19)^. In our study, there was no statistically significant difference between the groups regarding MN frequency in peripheral blood lymphocytes (p=0.239). It is suggested that genetic abnormalities are present in women with PCOS. In previous studies, the micronuclei frequency in the peripheral lymphocytes of patients with PCOS was reported to be increased^(6,20,21,22,23)^.

On the other hand, the MN frequency in cervical exfoliated cells was statistically significant between the groups (p=0.032). This study is the first to report on the genomic instability in cervical exfoliated cells of patients with PCOS. In the systematic review by Chittenden et al.^(24)^, it was shown that women with PCOS were more likely to develop endometrial cancer. It has recently been reported that there may exist an association between PCOS and breast cancer and PCOS and ovarian cancer^(25,26,27)^.

Cervical cancer is a significant health problem especially in developing countries. For prevention, early detection and treatment of preinvasive lesions that could progress to cervical cancer is very important. It has been shown exfoliated cervical cells of patients with moderate and severe dysplasia are observed with significantly higher frequency of MN compared with healthy women^(28)^. It may be suggested that the evaluation of the frequency of MN in exfoliated cervical cells may be helpful in establishing cervical cancer risk^(29)^.

### Study Limitations

Although the research has reached its aims, there were some unavoidable limitations. First, we had difficulty finding financial support for this research. For this reason, this study was conducted in a limited number of patients. Such studies should be carried out in wider series. Because of the same reason, we could not apply any additional test in assessing genotoxicity.

## CONCLUSION

According to our study results, although the study sample was small, there is a genomic instability in cervical exfoliated cells of women with PCOS. This study stands as the the first in the literature concerning cervical cytology and genomic instability. Patients with PCOS should be followed up for cervical cancer with more frequent intervals than healthy women.

## Figures and Tables

**Table 1:**
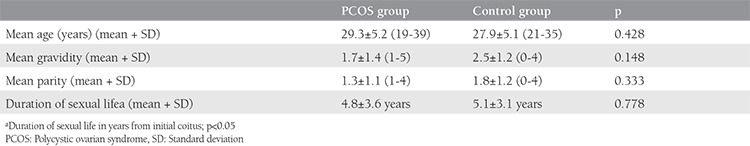
Mean age, mean gravidity, mean parity and duration of sexual life in patients with polycystic ovarian syndrome and the control group

**Table 2:**
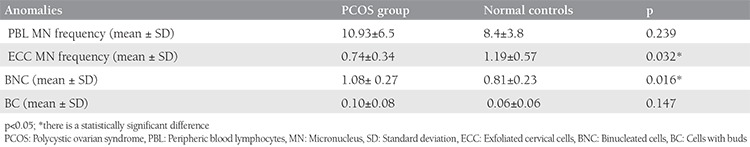
Average and standard deviation of the number of cells with micronuclei, binucleated cells, broken egg cells, and cells with buds
